# Immunotherapeutic Potential of Mollusk Hemocyanins in Murine Model of Melanoma

**DOI:** 10.3390/md22050220

**Published:** 2024-05-15

**Authors:** Emiliya Stoyanova, Nikolina Mihaylova, Nikola Ralchev, Silviya Bradyanova, Iliyan Manoylov, Yuliana Raynova, Krassimira Idakieva, Andrey Tchorbanov

**Affiliations:** 1 Department of Immunology, Stefan Angeloff Institute of Microbiology, Bulgarian Academy of Sciences, Acad. G. Bonchev Street, Block 26, 1113 Sofia, Bulgaria; stoyanova_e@microbio.bas.bg (E.S.); mihaylova_n@microbio.bas.bg (N.M.); nikola_ralchev@microbio.bas.bg (N.R.); silvybradyanova@microbio.bas.bg (S.B.); iliyanmanoylov@microbio.bas.bg (I.M.); 2 Institute of Organic Chemistry with Centre of Phytochemistry, Bulgarian Academy of Sciences, 1113 Sofia, Bulgaria; yraynova@orgchm.bas.bg (Y.R.); krasimira.idakieva@orgchm.bas.bg (K.I.)

**Keywords:** C57BL/6 mouse melanoma model, hemocyanins, anticancer therapy

## Abstract

The development of antitumor drugs and therapy requires new approaches and molecules, and products of natural origin provide intriguing alternatives for antitumor research. Gastropodan hemocyanins-multimeric copper-containing glycoproteins have been used in therapeutic vaccines and antitumor agents in many cancer models. Materials and Methods: We established a murine model of melanoma by challenging C57BL/6 mice with a B16F10 cell line for solid tumor formation in experimental animals. The anticancer properties of hemocyanins isolated from the marine snail *Rapana thomasiana* (RtH) and the terrestrial snail *Helix aspersa* (HaH) were evaluated in this melanoma model using various schemes of therapy. Flow cytometry, ELISA, proliferation, and cytotoxicity assays, as well as histology investigations, were also performed. Results: Beneficial effects on tumor growth, tumor incidence, and survival of tumor-bearing C57BL/6 mice after administration of the RtH or HaH were observed. The generation of high titers of melanoma-specific IgM antibodies, pro-inflammatory cytokines, and tumor-specific CTLs, and high levels of tumor-infiltrated M1 macrophages enhanced the immune reaction and tumor suppression. Discussion: Both RtH and HaH exhibited promising properties for applications as antitumor therapeutic agents and future experiments with humans.

## 1. Introduction

Each year, about 10 million patients worldwide are diagnosed with malignant tumors, and approximately one in six is lethal. Melanoma accounts for only 1% of skin cancers, but it remains the most aggressive. The transformation of melanocytes into tumor cells is characterized by the development of abnormal cell proliferation and metastasizing to different parts of the body [1,2]. Various types of treatment for patients with melanoma include surgery, chemotherapy, radiotherapy, immunotherapy, and targeted therapy. However, these therapies have many disadvantages, mainly due to their non-specific mechanism of action and toxicity to healthy tissues associated with severe side effects. Different types of cancer need specific therapy, which requires the search for new anticancer drugs [3,4,5].

The development of medicines from natural products has been enormously successful and has continued to be a key topic in the biological sciences in recent years. Biologically active natural products from plant, fungal, and marine sources exhibit specific mechanisms of action such as anti-inflammatory, antifungal, and anticancer effects [6,7,8].

Hemocyanins (Hcs) are the main glycoprotein components in the hemolymph of gastropods, whose main function is to carry oxygen throughout the tissues [9]. Their structure represents decamers or multi-decamers, consisting of ten subunits (330- to 550-kDa each) and comprising seven or eight globular functional units. Each unit contains two copper ions and can reversibly bind to one oxygen molecule. The molecular size of Hcs ranges from 3.3 to 13.5 MDa, which makes them the biggest proteins in nature. A number of studies have demonstrated that due to their xenogenic character, when injected into mammals, Hcs enhance the innate and adaptive immune response [10,11,12].

Biomedical interest in mollusk Hcs goes back more than 60 years, when these extracellular respiratory glycoproteins were first found to possess remarkable immunostimulatory properties in experimental animals and humans. Hc, isolated from the marine gastropod *Megathura crenulatea,* known as keyhole limpet hemocyanin (KLH), is one of the best-studied proteins. KLH is an announced golden standard and has been used for years for biomedical, immunological, and biotechnological applications [13,14,15].

Furthermore, other Hcs such as *Concholepas concholepas* (CCH), *Fissurella latimarginata* (FLH), and *Haliotis tuberculate* (HtH) have also been studied, showing similar or even better antitumor properties than KLH [16,17,18,19,20,21,22,23,24].

In our studies, we investigated the immunotherapeutic properties of two Hcs isolated from the marine gastropod *Rapana thomasiana* (RtH) and from the garden snail *Helix pomatia* (HpH) in a C-26 murine model of colon carcinoma [25,26]. The therapy of mice with induced colorectal carcinoma with both Hcs showed strong anticancer effects with variable efficacy depending on the treatment-regimen suppression of tumor volume growth, maintenance of high levels of antitumor antibodies, prolonged survival, and increase in certain populations of tumor-infiltrating lymphocytes. We demonstrated that cross-reactivity between both Hcs and C-26 carcinoma cells suggests a common tumor-associated epitope, and Sialyl Lewis X is a possible one. The same epitope has been found in the structure of both HpH and RtH but is much better represented in HpH [25,26].

*Helix aspersa*, commonly known as the garden snail, together with *Helix pomatia*, belong to the *Helicidae* family. Compared to numerous studies of structural organization, protein stability, and immunological properties of Hc isolated from *Helix pomatia*, the species *Helix aspersa* has been much less investigated [27,28]. Several studies claim that tissue lyophilisates from snails contain antioxidants important in the prevention of colorectal cancer. Extracts from tissues of *Helix aspersa* were shown to possess anticancer activity against breast cancer cells (Hs578T) [29,30]. Other assays with an extract from *Helix aspersa* showed high cytotoxicity against tumor cells by inducing necrosis and downregulating BcL2 expression [30].

In our previous study, we covalently linked the GD3 ganglioside-like mimicking peptide P4 to the Hc molecules of RtH and HaH and used them in different treatment schemes in a mouse model of melanoma. The administration of RtH-GD3P4 or HaH-GD3P4 conjugates suppressed tumor growth, prolonged the survival of treated animals, and generated tumor-specific cytotoxic T cells in the spleen. The therapy also induced tumor-infiltrating lymphocytes and generated significantly high levels of M1-type macrophages with dominant antitumor effects [31].

In the present study, we investigated the anticancer properties of native RtH and HaH analyzed in different application regimens in a mouse model of melanoma.

## 2. Results

### 2.1. RtH and HaH Purification

The process of isolation and purification of RtH and HaH was performed using pyrogen-free materials and reagents only. The presence of endotoxins in the final sterile Hc preparations was assessed by LAL assay and showed low values (4.5 EU/mg protein for RtH and 4.7 EU/mg protein for HaH).

### 2.2. Tumor Incidence, Tumor Development and Survival Analysis

**Figure 1:**
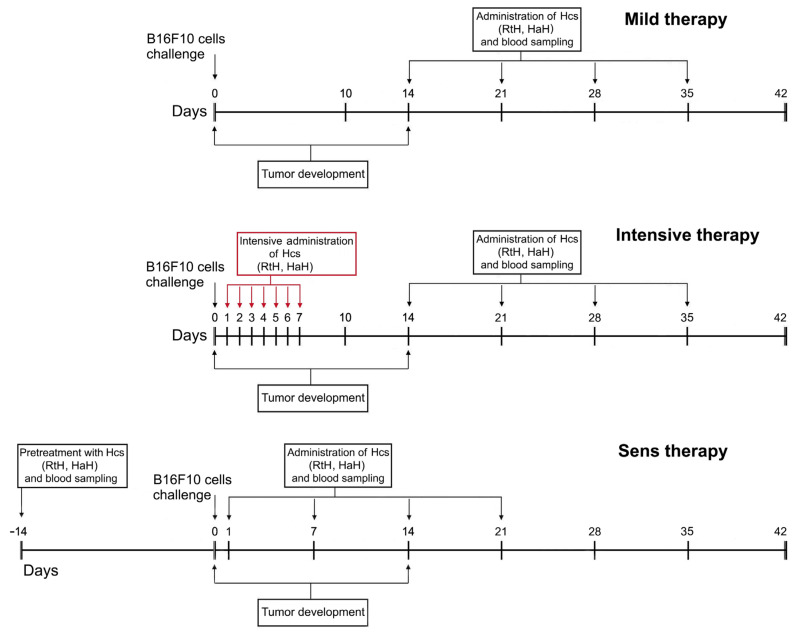
Therapeutic design and schedule for treatment. The challenge dose for all immunizations was 100 μg/mouse RtH or HaH.

The Hcs treatment and B16F10 cell challenge of animals of the respective groups are shown in Figure 1. In the control untreated group, the mice challenged with B16F10 cells developed palpable solid tumors on day 16 after the challenge.

**Figure 2:**
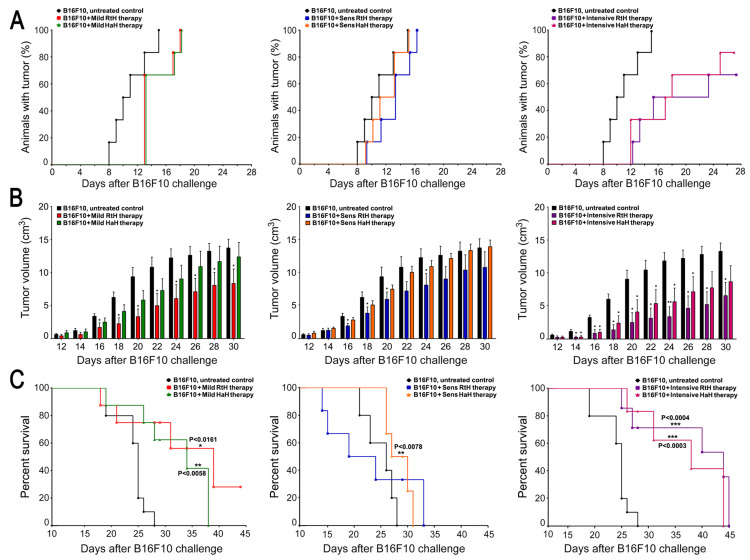
Development of tumors in experimental animals in three different therapeutic approaches with RtH and HaH. (**A**). Tumor incidence in the three different treatment schedules. The influence of Hcs therapy on tumor growth was followed in all experimental groups and compared with the control group. (**B**). Size of solid tumors in the considered therapeutic approaches. Tumor development was followed in all experimental groups and compared with the control group (injected with B16F10 cells, no therapy applied). Values in figures correspond to mean ± SD; *p* values were calculated using a two-way ANOVA test to determine the differences between any two groups (* *p* < 0.05, ** *p* < 0.01); (**C**). Survival analyses in different experimental groups (*n* = 10 mice each) in the B16F10 mouse melanoma model. Survival significance was determined via analysis of survival curves using the method of Kaplan and Meier, and the *p*-values were calculated (* *p* <0.05; ** *p* <0.01; *** *p* < 0.001) in comparison to B16F10-bearing mice. Representative data from three independent experiments are shown.

The administration of either RtH or HaH using all three therapeutic approaches showed a significant delay in tumor formation compared to the control group challenged with B16F10 cells without treatment (Figure 2B). Intensive therapy with RtH and HaH led to the strongest suppression of tumor growth and development, while the other two therapeutic approaches were less effective.

Similar results were obtained after monitoring the tumor incidences (Figure 2A). The mice treated intensively with both Hcs delayed tumor appearance, and 30% of animals from the RtH group and 20% from the HaH group did not develop solid tumors at the end of the observation (day 28). In addition, the other two regimens of Hcs administration exhibited a weak delay in tumor incidences compared to the control group, but all mice in the groups developed tumors during the period of observation.

With regard to survival, the best results were shown in the Mild treated group with RtH, and 30% of the mice survived at the end of observation (day 45 after the challenge with B16F10 cells), while the same challenge of the control group of animals was lethal and on day 28, the survival rate was 0%. The Mild treatment with HaH, as well as the Intensive therapy with either RtH or HaH, significantly prolonged the group survival by 10 and 15 days, respectively. The pretreatment with the two Hcs showed a weak prolonged survival compared to untreated controls with no survivors 33 days after the challenge with B16F10 cells (Figure 2C).

### 2.3. ELISA for Tumor-Specific IgM Antibodies

**Figure 3:**
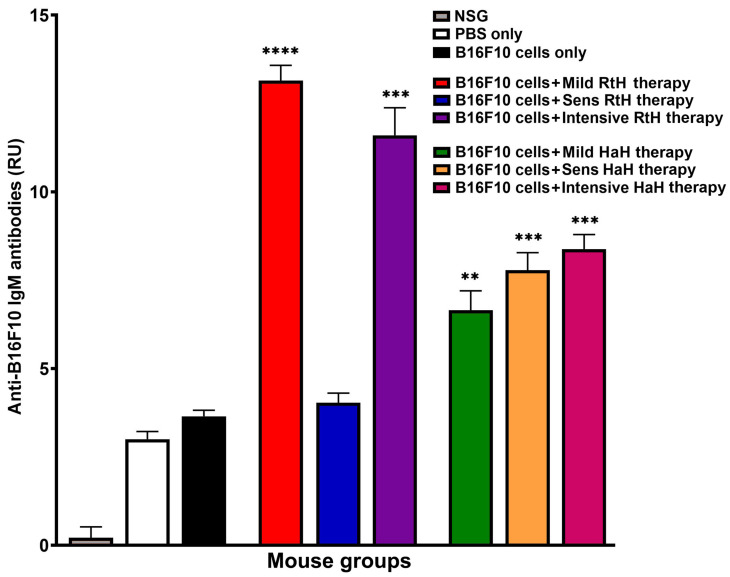
Detection of generated anti-B16F10 IgM antibodies in the experimental mice after being treated with RtH or HaH. Serum levels of IgM were measured by ELISA as described in the Section 4. The results are presented as relative units (RUs). All samples were triplicated, and average values were used for analysis. Mean ± SD values were calculated for each group (*n* = 6–14) using Dunnett’s multiple comparisons test (** *p* < 0.01; *** *p* < 0.001; **** *p* < 0.001), in comparison to control B16F10-bearing mice.

At the end-point of experiments, blood samples from all animals were collected from the retro-orbital sinus, and the mouse sera were tested individually by ELISA for anti-B16F10 IgM antibodies. The mice from all Hcs-treated groups developed anti-B16F10 IgM antibodies compared to the PBS-treated B16F10 tumor-bearing animals (Figure 3). The animals injected with RtH under Mild and Intensive regimens exhibited the highest significant values, while the anti-B16F10 IgM antibodies in mice pretreated with the same Hc and untreated control animals challenged with B16F10 cells recognize the B16F10 cell lysate loaded on the plates equally. The groups administered with HaH under all schemes of therapy showed significantly higher levels of anti-B16F10 IgM antibodies than the control group.

### 2.4. Impact on Splenocyte Proliferation by Hcs Ex Vivo

**Figure 4:**
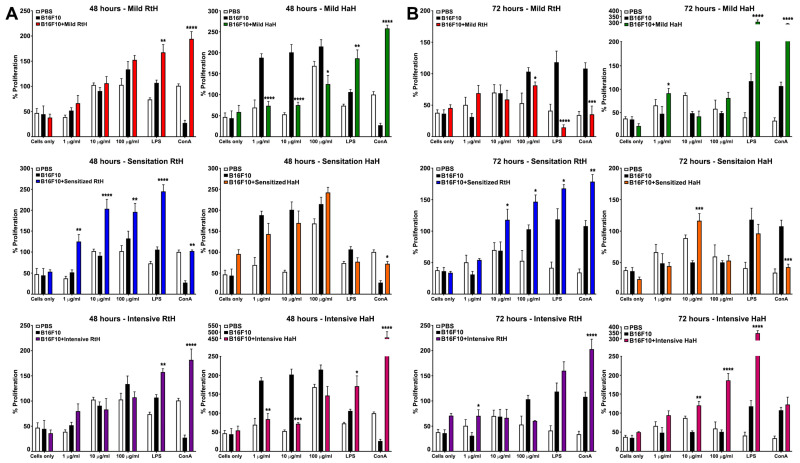
The effect of different Hcs concentrations (100, 10, and 1 μg/mL) on the proliferation of mouse splenocytes for 48 h (**A**) and for 72 h (**B**) incubation periods was determined by MTT assay. Results are expressed as the mean value ± SD of triplicated assays (*n* = 6–14); *p*-values were calculated using the Two-way ANOVA test (* *p* < 0.05; ** *p* <0.01; *** *p* < 0.001; **** *p* < 0.0001) in comparison to B16F10-bearing mice. Data are representative of four independent experiments.

The ability of the RtH and HaH to affect cell proliferation was studied ex vivo by adding different concentrations of both Hcs to cultured splenocytes from RtH- or HaH-treated B16F10 tumor-bearing animals or from the PBS-treated control mice injected with B16F10 cells. The MTT-based lymphocyte proliferation assay was evaluated by the addition of MTT for an extra 4 h after a 48 h or 72 h culture period and measuring the absorbance of converted dye. A dose-dependent increase in cell proliferation was observed in splenocytes isolated from mice under all regimens of RtH administration and in vitro stimulated with RtH for 48 h (significant for sensitized animals), compared to the splenocytes from the B16F10 tumor-bearing mice with the same ex vivo stimulation (Figure 4A, left panel). The highest values for cell proliferation were measured in the group with RtH-sensitized mice. In contrast, the ex vivo incubation of spleen cells isolated from HaH-treated mice under different schemes of administration with various amounts of HaH for 48 h suppressed cell proliferation compared to controls, with significance in the Mild- and Intensivetreated groups (Figure 4A, right panel).

Extended incubation with Hcs for 72 h exhibited the dynamic development and limits of the treatment effect. A significant dose-dependent increase in cell proliferation was measured in the animal group sensitized with RtH, as well as in mice intensively treated with HaH. Non-significant randomized values were found in the other groups after co-culturing with both Hcs (Figure 4B).

### 2.5. Cytokine Measures

**Figure 5:**
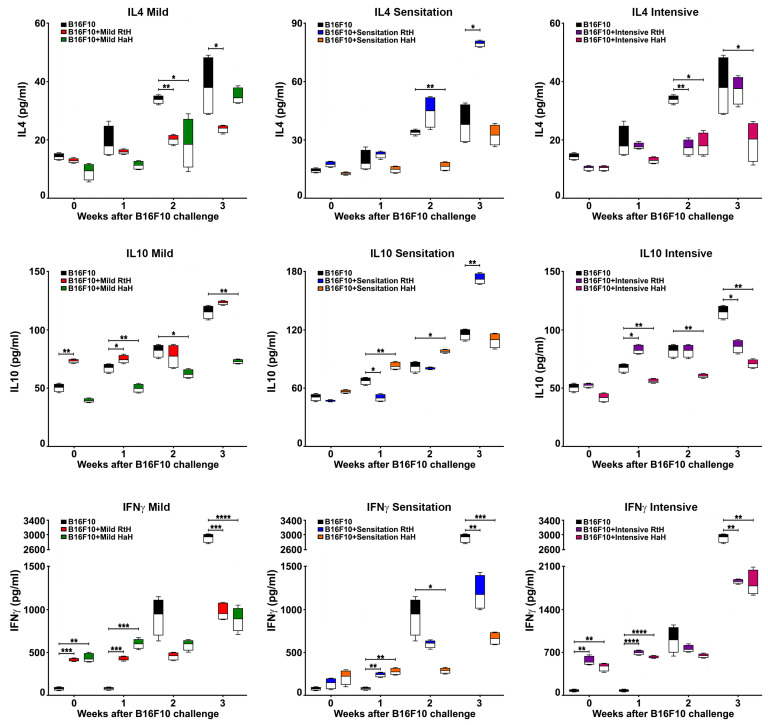
Treatment of B16F10-injected mice with RtH or HaH altered cytokine production. Serum levels of IL4, IL10 and IFNγ were measured by sandwich ELISA in all animal groups. All samples were triplicated, and the average values were used for analysis. The data are presented as mean ± SD for each group (*n* = 6–14); *p* values were calculated using the Two-way ANOVA test (* *p* < 0.05; ** *p* < 0.01; *** *p* < 0.001; **** *p* < 0.0001) in comparison to B16F10-bearing mice. Representative data from three independent experiments are shown.

During the tumor formation in B16F10 cell-challenged C57BL/6 mice, serum levels of several cytokines were monitored using ELISA to evaluate the effect of Hcs treatment: IL4, IL10, and IFNγ (Figure 5). As expected, tumor development in the control—PBS-injected B16F10 tumor-bearing animals—resulted in higher serum levels of IFNγ in the end-points compared to the Hcs-treated mice under all regimens of administration. The control group of mice also produced significantly higher concentrations of IL4 compared to mice with RtH and HaH therapy under Mild and Intensive administration, as well as IL10 compared to animals treated with both Hcs under the Intensive regimen.

The mice treated with RtH reached the highest levels of serum IL10 (Mild and Sensitized group) and IL4 (Sensitized group), while the administration of HaH never resulted in the highest values of monitored cytokines in any experimental animal group at the end of the observations.

### 2.6. Generation of CTLs

**Figure 6:**
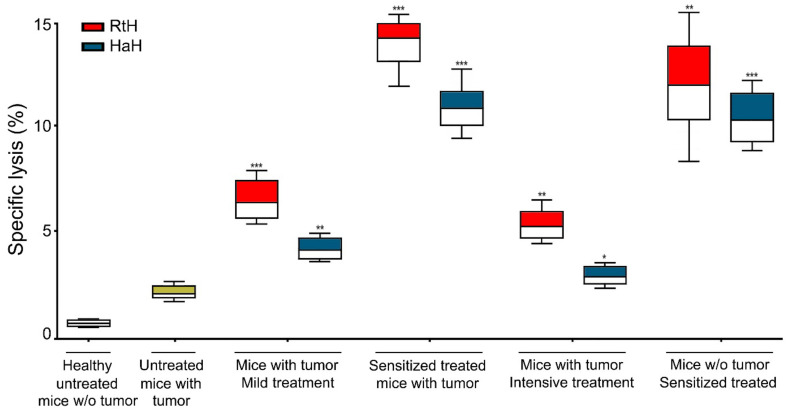
CTL activity of mouse splenocytes following immunization of tumor-bearing C57BL/6 mice with RtH or HaH. B16F10 melanoma target cells were incubated together with the effector splenocytes isolated from all treated mice. Resulted CTL activity was assessed as the concentration of lactate dehydrogenase released into the culture medium due to the target cell lysis. All samples were triplicated, and the data are presented as mean ± SD for each group (*n* = 6–14); *p* values were calculated using the two-way ANOVA test (* *p* < 0.05; ** *p* < 0.01; *** *p* < 0.001) in comparison to B16F10-bearing mice. Representative data from three independent experiments are shown.

The potential of RtH and HaH to induce CTLs specific to B16F10 cells under different regimens of therapy was evaluated by a specific CTL activity test. Isolated splenocytes from all experimental groups were used as effector cells against melanoma tumor B16F10 cells, and the released lactate dehydrogenase (LDH) after the specific CTL lysis of targets was measured. RtH administration to the B16F10 cell-challenged mice exhibited significantly higher results under all schemes of therapy compared to HaH-treated animals or PBS-injected B16F10 tumor-bearing mice (Figure 6). The highest values were measured in the animal groups sensitized with both Hcs, while surprisingly, the Intensive therapy induced a weak CTL response only in the test mice.

### 2.7. Phenotyping of Tumor-Infiltrated Cells

**Figure 7:**
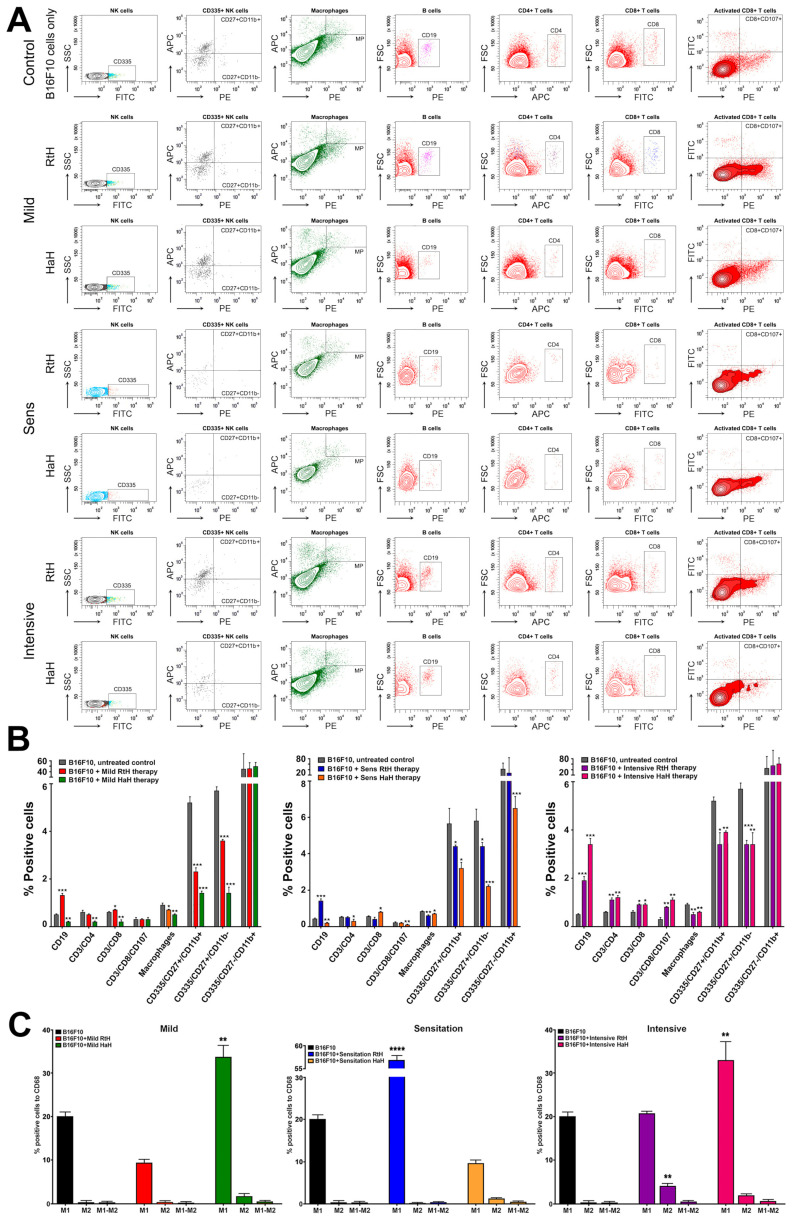
FACS analysis and phenotyping of lymphocyte infiltrates solid tumors after therapy with RtH and HaH under three different therapeutic approaches. Solid tumors were removed from the experimental animals and processed, and the resulting single-cell suspensions were incubated with a specific combination of labeled antibodies as described in the Section 4. Twenty thousand lymphocyte-gated cells from each tube were collected and analyzed by FACS. (**A**). Representative data from four experiments are shown. (**B**). The extracted results from all experiments are presented graphically as the percentage of total viable immune cells. (**C**). Graphical results of FACS analyzed tumor-infiltrating macrophages. Thirty thousand CD68-positive cells were analyzed from each sample for monitoring of M1/M2 discrimination as described above. The extracted results from all experiments are presented graphically as the percentage of total CD68-positive cells. The data are presented as mean ± SD for each group (*n* = 6–14); *p* values were calculated using the Two-way ANOVA test (* *p* < 0.05; ** *p* < 0.01; *** *p* < 0.001; **** *p* < 0.0001) in comparison to B16F10-bearing mice. Representative data of 3 independent experiments are shown.

To evaluate the effect of the immunization of C57BL/6 mice with RtH or HaH on the tumor microenvironment using the different therapeutic approaches of administration, a quantitative FACS analysis was performed. The tumors from all animals challenged by B16F10 cells were isolated and analyzed for the presence of various tumor-infiltrated lymphocytes (Figure 7A). In the RtH Mild-treated group, significant increases were found in the CD19^+^ B cells and CD8^+^ T cells compared to the untreated tumor-bearing animals, while significant decreases were observed in macrophages and NK cells in the early maturation stage (CD27^+^ CD11b^−^) and mature (CD27^+^ CD11b^+^) stage in the same group. In contrast, no increase for any cell type was found in the group Mild-treated with HaH, but significantly lower levels of CD19^+^ B cells, CD4^+^ and CD8^+^ T cells, macrophages, and NK cells in the early maturation stage (CD27^+^ CD11b^−^) and mature (CD27^+^ CD11b^+^) stage were observed compared to controls (Figure 7B, left panel).

Following the scheme with Hc pretreatment, a significant decrease in the percentage of cells was found in the specific cell populations, such as macrophages and NK cells in the Sensitized groups pretreated with RtH or HaH (Figure 7B, middle panel). Significantly decreased levels of CD19^+^ B cells, CD4^+^ and activated CD8^+^ T cells were also detected among the tumor-infiltrated lymphocytes after immunization with HaH compared to untreated tumor-bearing animals, while an increased number of B lymphocytes in the pretreated group and CD8^+^ T cells in HaH pretreated group was also found.

Statistically significant differences in the percentage of tumor-infiltrated lymphocytes were also found following the Intensive scheme of administration with both Hcs, which correlates with the best results for tumor incidence and survival, as well as the strong inhibition of the tumor growth in these mouse groups (Figure 7B, right panel). An increased number of cells were counted among the CD19^+^ B cells, CD4^+^, CD8^+^, and activated CD8^+^ T cells after immunization either with RtH and HaH compared to controls, while lower values were measured for macrophages and early mature and mature NK cells in the same animal groups.

### 2.8. Phenotyping of Tumor M1/M2 Macrophages

To assess whether both Hcs stimulate M1/M2 macrophage polarization in tumor tissue, the number of F4/80^+^ CD86^+^ (M1) and F4/80^+^ CD206^+^ (M2) was measured in a tumor single-cell solution by FACS (Figure 7C). The number of M1-like TAMs was significantly lower in the group with Mild therapy with RtH and equal to the controls in the Intensive group, while the Sensitization group exhibited much higher values than untreated tumor-bearing animals. In contrast, HaH treatment stimulated the polarization of significantly higher levels of M1 TAMs in the animal groups with Mild and Intensive therapy compared to mice challenged with tumor cells only, but this was not the case in the group with HaH pretreatment.

Significantly higher values for M2 TAMs were measured only in the group treated intensively with RtH compared to the control animals, while no significant changes in M2 levels were found among the TAMs in the other mouse groups treated with RtH or HaH. Low levels of double-positive M1/M2 macrophages were found in the groups of tumor-bearing mice treated with Hcs, as well as in the group with the tumor itself.

## 3. Discussion

Melanoma is a malignant tumor and represents the most aggressive type of skin cancer. It is responsible for the majority of skin cancer deaths, but the incidence and mortality rate of the disease differ widely across ethnic groups [1,32]. Leaving behind chemo- and radiotherapy, new biological therapies occupy advantageous positions due to the few side effects and specific targeting and effectiveness. Immunotherapy is based on the interaction between the immune system and target molecules on the surface of the cancer cell. Engaging several mechanisms of action, a number of monoclonal and chimeric antibodies have been approved in clinical trials for cancer treatment. At present, modern melanoma treatment includes monoclonal antibodies targeting the programmed cell death protein 1 receptor (PD-1) and its ligand (PDL-1), cytotoxic T-lymphocyte-associated protein 4 (CTLA-4), and lymphocyte-activation gene 3 (LAG-3), reactivating the lymphocyte arm from the adaptive immune system [33]. However, useful tools that provide highly effective tumor detection and destruction remain unavailable. Efforts to discover new molecules and mechanisms for successful melanoma therapy are ongoing.

Various natural products deliver huge numbers of new molecules that provide alternative mechanisms for the reactivation of the immune system for tumor recognition [7,8]. Among them are the Hcs, giant respiratory glycoproteins (molecular size up to 8–9 MDa) for oxygen transport isolated from mollusks and gastropods. They exhibit a wide range of beneficial immunological effects due to their potent Th1 stimulatory activity based on the composition of different carbohydrate residues that can reach up to 9% of the molecular weight. The Hcs have been approved as potent protein carriers for antibody production and a key component of anticancer therapeutic vaccines [11,31,34].

By themselves, Hcs are highly immunogenic due to the specific pattern of oligosaccharide composition, the most common being mannose and fucose. The natural ligands of these oligosaccharides are the mannose receptor, a member of the C-type lectin receptors family, and Toll-like receptor 4 (TLR4). These receptors are highly expressed on the surface of many cell types, part of innate immunity, and are involved in the activation of murine APCs (dendritic cells and macrophages), together with NK cells and mononuclear cells [35]. Such an activation results in increased expression levels of costimulatory molecules (CD80, CD86, CD40, CD83, and HLA-DR), as well as secreted interleukin 10 (IL10), IL12, IL6, tumor necrosis factor-α (TNF-α), IL12p40, and IL23α. Keyhole Limpet Hemocyanin (KLH), isolated from the marine gastropod *Megathura crenulata*, is a golden standard for Hcs exploring and applications. Another two Hcs have been isolated from the Pacific mollusk *Concholepas concholepas* (CCH) and from the Chilean mollusk *Fissurella latimarginata* (FLH). Using the B16F10 mouse model of melanoma and treatment with KLH, CCH, or FLH under the same schedules, a variety of immune responses against the tumors were found due to the different content of oligosaccharides [17]. Among them, FLH exhibits the strongest immunogenicity and antitumor activity against melanoma in the B16F10-induced mouse model in vivo, suppressing tumor growth and prolonging the survival of mice. In our studies, we used several Hcs for the treatment of different tumors in various animal models under three generated schemes of treatment. Two Hcs, isolated from marine gastropod *Rapana thomasiana* (RtH) and garden snail *Helix pomatia* (HpH), were used for therapy using a C-26 murine model of colorectal carcinoma based on the cross-reactive tumor-associated epitope Sialyl Lewis x [25,26]. The therapy of tumor-bearing mice with Hcs leads to the generation of a combined immune response, including high levels of tumor-specific antibodies, the secretion of proinflammatory cytokines, and CTL. The effectiveness of Hcs administration is manifested in the suppression of tumor growth and the prolonged life of treated mice and is highly dependent on the treatment regimens (Mild, Intensive, and Priming) and the selected Hc [25,26].

For therapy using a B16F10 murine melanoma model in C57BL/6 mice, we used two Hc—RtH and a new one, isolated from the terrestrial snail *Helix aspersa* (HaH)—as a base of conjugated vaccines, containing ganglioside mimotope GD3P4 peptide. Both protein-engineered vaccines (RtH-GD3P4 and HaH-GD3P4) exhibited a strong antitumor immune response with decreased tumor incidence and suppressed tumor growth, and they prolonged the survival of treated animals, depending on the different regimens of therapy [31].

In the present study, we used the same Hcs (RtH and HaH) alone in order to follow their independent anticancer effect in the B16F10 murine melanoma model. Using three different schemes of immunization, both Hcs induced tumor growth suppression, delayed tumor incidence, and increased the survival of treated animals. It was important to discover the mechanism of action of Hcs and the way in which different immunization regimens affect tumor behavior.

Two types of IgM antibodies are involved in recognizing and removing tumor cells: natural antibodies, which are present in the serum before tumor development and are part of unspecific first-line defense, and adaptive antibodies, generated after tumor antigen stimulation. Natural and adaptive IgM antibodies exhibited a potential to eliminate cancer cells by recognition of tumor-modified cell surface neo-antigens developed during tumorigenesis and to apply a direct cytotoxic effect to tumor cells by activating the complement system [36,37]. The generation of significant high titers of anti-B16F10 IgM antibodies in the groups of mice immunized with both Hcs would have beneficial antitumor effects in vivo as they recognize surface antigens on B16F10 cells. Together with the increased proliferation activity of splenocytes in Hc immunized groups, RtH showed better antitumor properties according to the obtained results.

Significantly increased IL4 production was observed in the control tumor-bearing group of animals during the follow-up period. High levels of IFNγ, in combination with low levels of IL4 in sera of Hc-treated mice, suggest a switch to classically activated M1 macrophages and cytotoxic T cells. These antitumor responses are characterized by limiting disease progression [35,38]. Significant differences in IL-10 secretion were observed between the Hc-treated and the untreated control groups, as the high level of this tolerogenic cytokine suggests tumor progression.

Both Hcs induced immune cell infiltration into the tumors, which participated in the generation of CTLs. The three schemes of treatment showed a randomized advantage of RtH or HaH due to the different cell infiltration values. Even if the levels of IFNγ are not very high in the sera of Hc-treated mice, the local intra-tumor IFNγ production by tumor-infiltrated CD4 Th1, CD8 cytotoxic T lymphocytes, and NK cells induced the generation of specific antitumor CTLs, responsible for tumor growth suppression. Here, again, RtH had better properties for the stimulation of high levels of CTLs, compared to HaH.

Positive NK cells can be categorized into three subgroups based on the surface expression of CD27 and CD11b: CD27-hi/CD11b-lo (immature), CD27-hi/CD11b-hi (mature), and CD27-lo/CD11b-hi (senescent) cells, which have different phenotypic and functional properties. When RtH and HaH were administered, lower percentage values were found in all three therapeutic approaches compared to the untreated control group. Only the CD27^−^ and CD11b^+^ populations showed similar values in the Intensive and classical groups when compared to the percentages obtained in untreated animals.

Both M1 and M2 macrophages produced specific cytokine profiles depending on tissue localization and the microenvironment. Therefore, the M1/M2 classification is based on general characteristics and does not exclude overlapped phenotypes of macrophages depending on their tissue location. Different M1/M2 hybrid phenotypes during the antitumor response have been published, and each combination may play a specific role in the tumor microenvironment [39].

The administration of both Hcs and the tumor itself induced high numbers of tumor-removing M1 macrophages. Furthermore, HaH had an advantage for the generation of significantly higher levels of tumor-infiltrated M1 macrophages under the Mild and Intensive regimens of treatment, compared to RtH, while the last one exhibited better properties under the Sensitization scheme of administration. Not surprisingly, both Hcs and the tumor itself also induced low levels of M1/M2 hybrid phenotypes.

The histological investigation of the solid tumors can also provide additional information about the ways in which the Hcs treatment resulted in the suppression of tumor growth. The histological investigation of the solid tumors can also provide additional information on how the Hcs treatment resulted in a suppression of tumor growth (Figure S1). Cells deprived of oxygen and nutrients die and form pyknotic nuclei and necrotic patches. The presence of immune cell infiltrates along with the formation of apoptotic bodies in the groups treated with Hcs are the positive features of administered therapy.

Adipocytes in the surrounding area of cancer cells, called cancer-associated adipocytes (CAA), showed phenotypic and functional changes. They acquire characteristics different from those of naïve fatty tissue cells, such as smaller sizes, irregular fibroblast-like shapes, small lipid droplets, and an increased level of inflammatory cytokines. Moreover, cancer-associated adipocytes can undergo conversion to cancer-associated fibroblasts, which further modifies the tumor microenvironment and favors melanoma progression. Although most of the research characterizing CAA comes from studies focused on the biology of breast cancer, it is now recognized that when tumor cells invade the surrounding adipose tissue, adipocytes disappear, and fibroblast-like cells accumulate in all tumors growing in a microenvironment dominated by adipose tissue (such as stomach, breast, colon, kidney, prostate and ovarian cancer, and melanoma). Keeping in mind the importance of adipocytes in other types of cancer, we consider that these subcutaneous adipocytes may promote melanoma growth and progression [40]. Such understandings may explain the visible changes in the histology of mouse skins in the tumor region in the groups treated with both Hcs. Additional studies are needed to investigate how the remodeling of adipocyte-layer morphology affects tumor growth and melanoma progression.

The obtained results from the treatment with both Hcs under the different regimens are not unidirectional. There are a considerable number of factors that influence the various pathological indicators. As expected, the use of large macromolecules such as Hcs always leads to different results between different treatment regimens. The treatment of a mouse melanoma model with RtH and HaH, conjugated to GD3-TACs [31]; CCH and FLH, conjugated to GD2-TACs [34], CCH and FLH [17]; and a treatment of mouse colon carcinoma model with RtH and HpH [25,26] showed a changed dominance of positive immune response to different Hcs and different regimens of treatment concerning the pathological indicators, without a conclusion of the best approach. The complexity of the immune response implies variation and has always varied; therefore, the aim of the present results is to demonstrate the properties of these huge proteins as anticancer agents.

Both Hcs exhibited a strong anticancer effect after the immunization of B16F10-challenged mice. The successful use of RtH and HaH for antitumor therapy depends on the immunization schedules, providing promising results and the potential for extended human experiments and clinical trials.

## 4. Materials and Methods

### 4.1. Antibodies

Anti-mouse Phycoerythrin (PE)-conjugated F4/80, CD107a, CD68, and CD27; Allophycocyanin (APC)-conjugated CD4, CD86, and CD11b; Brilliant Violet 421-conjugated CD163; Fluorescein isothiocyanate (FITC)-conjugated CD8 and CD335; Pacific Blue-conjugated CD19; and eFlour450-conjugated CD45 and CD3 mAbs (eBioscience, Frankfurt, Germany) were used for fluorescence-activated cell sorting (FACS) experiments. Alkaline phosphatase (AP)-labeled anti-mouse IgG or anti-mouse IgM Abs (Sigma-Aldrich, Taufkirchen, Germany) were used for enzyme-linked immunosorbent assay (ELISA).

### 4.2. Cell Line

Murine melanoma cell line B16F10 (ATCC^®^ CRL6475 ™) was kindly provided by Dr. Sergej Tomic, Institute for the Application of Nuclear Energy (INEP), University of Belgrade. The cells were cultured at 37 °C/5% CO_2_ in complete Dulbecco’s Modified Eagle Medium (DMEM, Gibco, Gaithersburg, MD, USA) supplemented with 10% heat-inactivated fetal calf serum (FCS), 1 mM sodium pyruvate, 4 mM L-glutamine and antibiotics. The monocellular suspension was prepared from an 80% confluent cell monolayer by accutase (eBioscience) and cell strainers (BD Biosciences, Erenbodegem, Belgium).

### 4.3. B16F10 Cell Lysate Preparation

B16F10 cell lysate was prepared as previously described [31].

### 4.4. Animals

Female C57BL/6 mice were purchased from The Jackson Laboratory (Bar Harbor, ME, USA). The 6-week-old animals were housed at 22 °C with a light/dark cycle of 12/12 h under specific pathogen-free (SPF) conditions in a barrier-type animal house at the Institute of Microbiology, Bulgarian Academy of Sciences. All animal experiments and manipulations were approved by the Animal Care Commission at the Institute of Microbiology (N286/16.04.2021) in accordance with national regulations and the Guidelines for the Care and Use of Laboratory Animals of the European Union (EU Directive 2010/63/EU).

### 4.5. Isolation and Purification of RtH and HaH

The purification and isolation of RtH and HaH from the hemolymph of *Rapana thomasiana* or of *Helix aspersa*, respectively, were performed as previously described [21,27]. Further, both Hcs were subsequently purified by gel filtration chromatography and passed through a Detoxi-Gel column (Detoxi-Gel column, Thermo Fisher Scientific, Rockford, IL, USA) for endotoxin removal. The final concentrated Hc solutions were tested for residual endotoxins using Limulus Amebocyte Lysate coatest gel (LAL) (Chromogenix AB, Molndal, Sweden) under pyrogen-free conditions.

### 4.6. Mouse Model of Melanoma and Treatment Schedule

Female 10-week-old C57BL/6 mice were randomized and divided into seven groups (thirty animals per group). Ten mice from each group were set aside for survival analysis, and the other animals were all used for ex vivo tests.

The animals were challenged subcutaneously (s.c.) into the right flank with a single cell suspension from B16F10 murine melanoma cells (1.5 × 10^5^ cells/mouse), and after the formation of a palpable solid tumor, two groups of mice were immunized once a week with 100 μg/mouse RtH (Mild RtH group) or HaH (Mild HaH group) intratumorally for 4 weeks (Figure 1).

Two more animal groups were treated intensively once daily by the administration of the same quantity of RtH or HaH by s.c. injection in the tumor cells’ inoculation area for 7 consecutive days (Intensive RtH and Intensive HaH groups), beginning the next day after the B16F10 cell challenge. The therapy continued with weekly intratumoral injections of the same amounts of RtH or HaH for 4 weeks.

Another two groups of mice started the therapy with sensitization with 100 μg/mouse of RtH or HaH 14 days prior to the B16F10 cell challenge (Sensitized RtH and Sensitized HaH groups). Later, the mice were immunized once weekly with the same amounts of RtH or HaH by s.c. injection in the area of tumor cells’ inoculation for 4 weeks, starting the next day after the challenge with B16F10 cells.

Two control groups of animals were treated with PBS only with or without the B16F10 cell challenge. RtH or HaH was administered to another two control animal groups without a B16F10 cell challenge under the scheme of Sensitized treatment. The mice from all groups were bled weekly from the retro-orbital sinus, and the sera were stored frozen at −70 °C for further analyses.

### 4.7. Tumor Assessment and Organ Collection

Tumor incidence and growth, as well as survival, were evaluated as previously described [32], and the tumor volumes were calculated using the formula:

Volume (cm^3^) = width^2^ × length × 0.52

The animal groups were monitored for 6 weeks, and the survival of the treated groups was compared with the survival rates of the B16F10-cell-challenged group without treatment.

Duplicated groups of experimental animals were sacrificed on day 25 after the challenge with B16F10 cells, and the solid tumors and spleens were collected from all mice.

### 4.8. ELISA for Determination of IgM Antibodies against B16F10 Cells

The terminal animals from all duplicated groups were bled on the 25th day after the challenge with B16F10 cells, and the mouse sera were tested for IgM antibodies recognizing B16F10 cells. Then, 96-well plates (Nunc, Roskilde, Denmark) were loaded with B16F10 cell lysate (250 µg/mL) and incubated overnight at 4 °C. After extensive washing with PBS/0.05% Tween 20, the plates were blocked with 1% bovine serum albumin (BSA) in PBS/0.05% Tween 20 for 2 h at room temperature (RT). Next, the plates were washed again and incubated with the diluted serum samples (50-fold in PBS/0.05% NaN_3_) for 1 h at RT, washed again, and incubated with an anti-mouse IgM antibody conjugated with alkaline phosphatase for 1 h at RT. After washing, PNPP (p-Nitrophenyl Phosphate, Disodium Salt) (Sigma-Aldrich) was added, and the plates were read at 405 nm. Sera from untreated C57BL/6 and immunodeficient NOD-*scid IL2r*γ^null^ (NSG) mice were used as negative controls. The results obtained are presented as relative units (RUs) corresponding to the dilution of standard antibodies recognizing B16F10.

### 4.9. Proliferation Assay

Spleens from C57BL/6 mice from all duplicated experimental groups, sacrificed on day 25 after the challenge with B16F10 cells, were removed and ground through sterile cell strainers (BD Biosciences, Erenbodegem, Belgium) for monocellular suspension preparation. After the lysis of the erythrocytes with a hypotonic ammonium chloride solution, the cells were counted by a haemocytometer. Isolated splenocytes were cultured (2 × 10^6^ cells/mL) in complete colorless RPMI (Roswell Park Memorial Institute) 1640 medium (GE Healthcare, Hatfield, UK) containing 10% FCS (fetal calf serum), 4 mM L-glutamine, and antibiotics. The cells were cultured in the presence of different concentrations of either RtH or HaH (100 μg/mL, 10 μg/mL, and 1 μg/mL) in 96-well cell culture plates for 48 h or 72 h at 37 °C/5% CO_2_.

The control cells were stimulated either with 10 μg/mL lipopolysaccharide (LPS) (from *E. coli*, Sigma, L-2630) or 10 µg/mL concanavalin A (ConA) (Sigma) or were cultured in medium only. MTT (3-[4,5-dimethylthiazol-2-yl]-2,5-diphenyltetrazolium bromide; thiazolyl blue) was added (5 mg/mL) for an additional 4 h. Later, the supernatant was removed and 150 μL/well DMSO (dimethyl sulfoxide) was used to dissolve the formazan crystals. Proliferation was evaluated by measuring the absorbance at a wavelength of 590 nm corrected by subtracting the absorbance at 620 nm.

### 4.10. Cytokine Detection

Interferon gamma (IFNγ), interleukin 4 (IL4), and interleukin 10 (IL10) levels were measured in mouse sera using ELISA sets (Abcam, Cambridge, UK) according to the manufacturer’s instructions.

### 4.11. Cytotoxicity Assay

The level of generated B16F10 cell-specific cytotoxic T lymphocytes (CTLs) was evaluated by a non-radioactive cytotoxicity assay kit (CytoTox, Promega, Madison, WI, USA) as previously described [31]. Briefly, cultured murine melanoma cell line B16F10 was detached from a flask by enzymes and transferred to a 96-well culture plate (1 × 10^4^ cells/well). The experimental animals were sacrificed on the 25th day after the B16F10 cell challenge, and the splenocytes were isolated from all mice as described above (see the Section 4.9). These splenocytes were used as effector cells in a cytotoxic assay and were added to the wells (4 × 10^5^ cells/well) in a ratio of 1:40 with the target B16F10 cells. The specific lysis (%) was evaluated according to the cytotoxicity assay kit instructions.

### 4.12. Analysis of Tumor Infiltration

The phenotype of intratumor cell infiltration was determined by FACS analyses. Briefly, single-cell suspensions from all solid tumors were incubated with a variety of anti-mouse antibody mixes to follow the respective cell populations: CD19^+^ CD4^+^ CD8^+^, CD3^+^ CD8^+^ CD107^+^, F4/80^+^, CD335^+^ CD27^+^ CD11b^+^, CD335^+^ CD27^+^ CD11b^−^ and CD335^+^ CD27^−^ CD11b^+^. The phenotype of tumor-infiltrated lymphocytes was analyzed with a BD LSR II flow cytometer using Diva 6.1.1. software (BD Biosciences, Mountain View, CA, USA).

### 4.13. M1 and M2 Macrophage Phenotyping

We used flow cytometry analysis to discriminate M1 and M2 types among the tumor-associated macrophages (TAMs), as already described [31]. The obtained single-cell suspensions (see above) were washed with FACS buffer and distributed into the tubes. The samples were incubated with either CD68-PE/CD86-APC or CD68-PE/CD163-Brilliant Violet 421 anti-mouse antibodies. The gated CD68-positive cells were analyzed for M1 and M2 macrophage phenotypes with a BD LSR II flow cytometer using Diva 6.1.1. software.

### 4.14. Statistical Analysis

All statistical analyses including survival significance were performed with Prism 10 software from GraphPad (San Diego, CA, USA). The two-way ANOVA test was used to determine differences between the two groups, and values in the figures correspond to mean ± SD. All ELISA, cytokine, and cytotoxicity samples were triplicated. Survival significance was determined using the method of Kaplan and Meier. A value of *p* < 0.05 was considered statistically significant.

## Data Availability

The data presented in this study are available on reasonable request directed to the corresponding author.

## Supplementary Materials

The following supporting information can be downloaded at: https://www.mdpi.com/article/10.3390/md22050220/s1, Figure S1: Histological tumor and skin analyses. A. Tumor sections stained with haematoxylin/eosin. Scale bar, 50 µm, original magnification x 100; B. Skin sections stained with haematoxylin/eosin. Scale bar, 50 µm, original magnification x 100; Data are representative of 10 mice per group.
