# Supplementary material for: Immunotherapeutic Potential of Mollusk Hemocyanins in Murine Model of Melanoma
Source: Mar Drugs. 2024 May 15;22(5):220. doi: 10.3390/md22050220 (PMC11122751; doi:10.3390/md22050220)
Supplement: Supplementary file 1 [file marinedrugs-22-00220-s001.zip › marinedrugs-2950383-supplementary.pdf]

## Supplemental data

## Materials and Methods

*Histological analysis of tumors and skin*

The groups with experimental mice immunized with RtH or HaH following the different schemes of treatment were sacrificed by cervical dislocation 28 days after tumor cell inoculation. The isolated tumors and skin were then fixed in 4% phosphate-buffered formalin (pH 7.4) for 14 days. After the fixation, the samples were washed, embedded in paraffin (Paraplast Plus®, Sigma-Aldrich), and sectioned. The sections (6 mm) were deparaffinized in xylene substitute (Tissue-Tek® Xylene, Sakura Finetek, CA, USA). The preparations were rehydrated using a descending alcohol series, then the slides were stained using a standard hematoxylin and eosin (H&E) staining protocol. After washing with water, the slides were dehydrated using an ascending alcohol series and xylene substitute, and then dried. Finally, the coverslips were mounted using a Vitro-Clud embedding medium (R. Langenbrinck, Emmendingen, Germany). The preparations were analyzed by light microscopy using a Leica DM2000 (Wetzlar, Germany), and the resulting images were processed with Leica Application Suit X 3.7.4.23463.

## Results

*Monitoring the effect of Hcs treatment on the pathohistological changes of the solid tumors and skin as a result of B16F10 cells challenge*

The observation of tumor sections after staining with hematoxylin/eosin, revealed the characteristics of rapidly developing tumor formations - pyknotic nuclei and necrosis surrounded by cancer cells. The observed pyknotic nuclei are small condensed nuclei from apoptotic cells which appear as a result of rapid division and growth of tumor cells, while the rate of formation of new blood vessels in the tumor remains slower. Areas of immune cell infiltrates attracted by the inflammation caused by the dying cancer cells are also observed. The tumors from the control untreated group of animals showed specific features such as nuclear hyperchromasia and had a lower presence of apoptotic cells (Fig. S1A).

Different forms of tumor necrosis could be a feature of those that have an aggressive growth pattern. Initial necrosis has a specific morphology characterized by well-defined foci of necrosis with granular nuclear and cytoplasmic debris observed in animals Intensively treated with RtH. The Sensitization group with HaH therapy showed pyknotic nuclei, reduced nuclear/cytoplasmic ratio, and nuclear degradation. The same therapy of mice with RtH showed large blood vessels, focal areas of necrosis, and the formation of apoptotic bodies. In the groups treated with the classical therapy with both hemocyanins, infiltrates and scattered cells with pyknotic nuclei and apoptotic bodies were observed.

Histology of skin sections showed that hair follicles in the dorsal skin of mice progressed to the anagen phase of the second cycle of hair growth, while animals Intensively treated with RtH remained in the first telogen

phase (Fig. S1B). We noticed adipocyte layers (black arrows) immediately adjacent to the tumor were reduced in size compared to this further away, consistent with tumor-induced lipolysis. The Hc therapy of mice with tumors introduced dramatic changes in the skin histology in the tumor area, especially various HaH-based therapies.

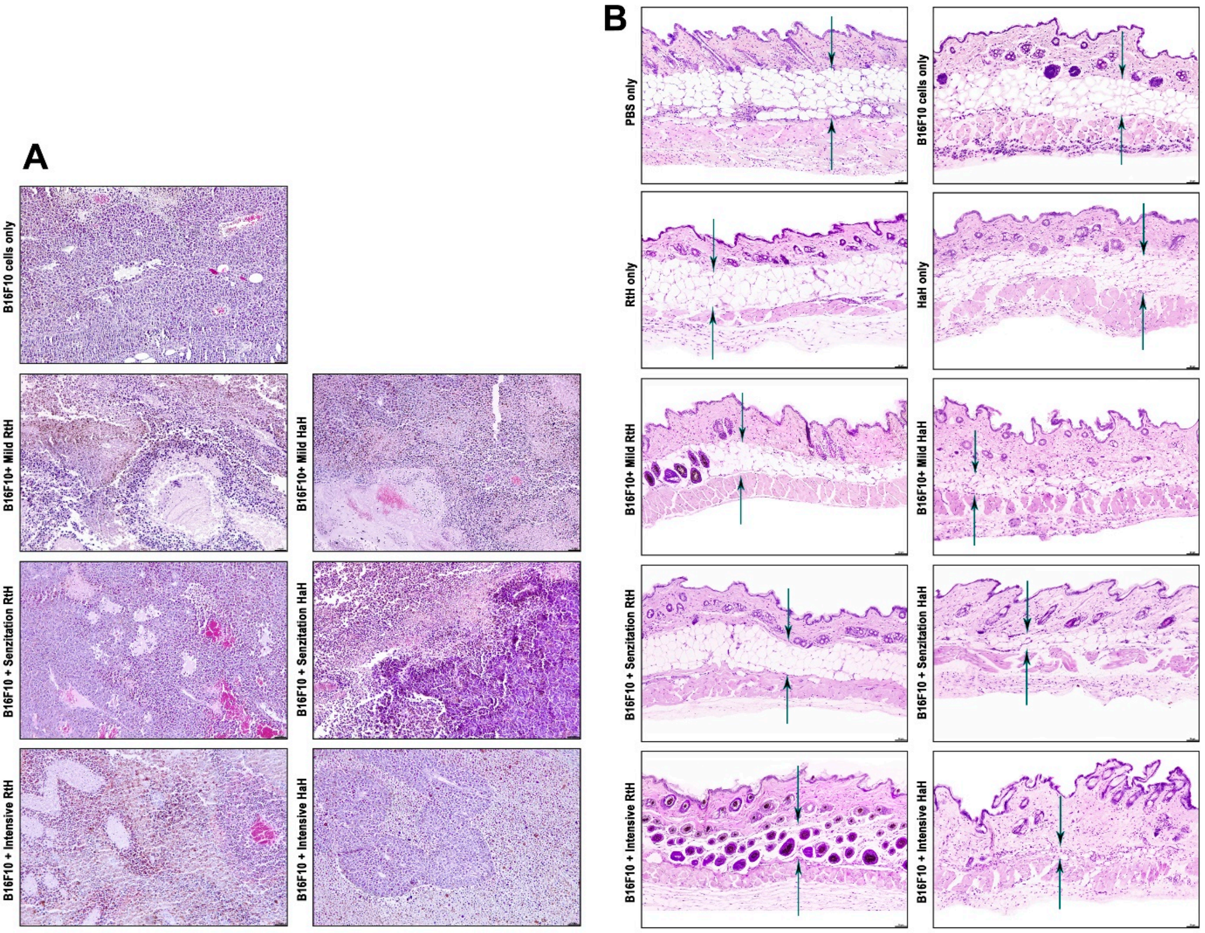


**Figure S1.** Histological tumor and skin analyses. **A.** Tumor sections stained with haematoxylin/eosin. Scale bar, 50 μm, original magnification x 100; **B.** Skin sections stained with haematoxylin/eosin. Scale bar, 50 μm, original magnification x 100; Data are representative of 10 mice per group.
